# Supplementary material for: Accuracy of HemoCue301 portable hemoglobin analyzer for anemia screening in capillary blood from women of reproductive age in a deprived region of Northern Peru: An on-field study
Source: PLoS One. 2023 Nov 14;18(11):e0293984. doi: 10.1371/journal.pone.0293984 (PMC10645325; doi:10.1371/journal.pone.0293984)
Supplement: Attachment — Submitted filename: COMMENTS 2.pdf [file pone.0293984.s002.pdf]

## COMMENTS

**Study Title**: "Accuracy of the portable Hemoglobin Analyzer HemoCue301 in Women of Reproductive Age in a Deprived Region of Northern Peru: An On-Field study"

### Summary/observation

The study is very significant as it addresses an important issue in public health. The study aimed to assess the suitability and accuracy of a portable device called HemoCue301 which is used to classify anemia. While the study appreciates the use of HemoCue301, it advised that, the used of the device for the classification of iron deficiency anemia (IDA) should be done with caution.

### Introduction

1. Authors should kindly take note and proofread the writing. Example, authors may take a second look at line 56 and 57 of page 3

### Method

2. No justification was provided for the exclusion of heavy smokers from the study, line 82 page 5.
3. How were the blood samples transported to the laboratory where automated analyzer was used? Line 90 to 96 of page 5
4. Quality control issues were not indicated
