# Supplementary material for: Accuracy of HemoCue301 portable hemoglobin analyzer for anemia screening in capillary blood from women of reproductive age in a deprived region of Northern Peru: An on-field study
Source: PLoS One. 2023 Nov 14;18(11):e0293984. doi: 10.1371/journal.pone.0293984 (PMC10645325; doi:10.1371/journal.pone.0293984)
Supplement: Attachment — Submitted filename: PONE-D-23-21513 REVIEWER REPORT.docx [file pone.0293984.s001.docx]

REVIEWER REPORT FOR MANUSCRIPT PONE-D-23-21513

**General comment**

Alarcón-Yaquetto et al have attempted to add data to the venous vrs capillary haemoglobin comparison study viz-a-viz different devices/instrument conundrum. However, the authors have not clearly defined basic concepts well to enable the reader to properly situate the findings. For example, the title is a bit misleading considering the study design employed. In line 86 – 88 (page 5), the authors write that “Briefly, 301 cuvettes were filled with a drop of blood from the middle finger fingertip. The first 2 drops of blood were discarded. The microcuvette was put in the cuvette holder and a measurement was done immediately”. In line 90 – 91 (page 5), the authors write that “Upon recruitment, a trained phlebotomist took venous samples of each woman with the BD vacutainer system”. The laboratory samples were venous blood samples. Therefore, the study compared venous haemoglobin (from automated analyser) to capillary haemoglobin (Haemocue 301). it is a known fact that venous haemoglobin and capillary haemoglobin are not equivalent (<https://www.mdpi.com/2075-4418/12/12/3191>; [10.1371/journal.pone.0278350](https://doi.org/10.1371%2Fjournal.pone.0278350)). The title of the study is thus misleading and should be modified to reflect exactly what was done. Again, the distinction between iron deficiency anaemia and anaemia has not been made; the authors give the impression that the two terms are synonymous. However, that is not a scientific fact. This has confounded the write up and the data analyses as well as its interpretation. The manuscript should thus be revised holistically to address these.

**Abstract**

1. The abstract has been written on the basis of the assumptions that were used to analyse the data. As suggested above and below, the abstract need a re-work after taking the suggested revisions into consideration.
2. In the objective statement, indicate that the sampling site was different for the Haemocue and the automated haematology analyser.

**Introduction**

1. Line 40 – 41, authors seem to portray IDA as being equivalent to anaemia by the statement that “Peru is one of the countries where IDA is a severe public health problem as shown by national statistics.”
2. Line 56 – 57, authors write “Handheld devices have been routinely used to screen for IDA in LMIC countries such as the HemoCue system” to imply that Hemocue is a device for undertaking iron studies. This is misleading since the device estimates haemoglobin levels which is different from ferritin, iron, TIBC etc.
3. Authors again write that “The main objective of this study is to assess the suitability and accuracy of this device as a method for IDA diagnosis in women of reproductive age in a coastal city with resource constraints” Hemocue is not meant for iron studies.

**Exclusion criteria**

1. When authors write “Exclusion criterion was amenorrhea non explained by pregnancy and heavy smoking” does this mean that both conditions should be present before exclusion?

**Statistical analyses**

1. Authors defined Microcytosis using the CBC reported mean corpuscular values (MCV) < 80 fl.,
2. and hypochromia by mean corpuscular hemoglobin (MCH) values below 27. There are many causes of microcytic-hypochromic red blood cell picture. With the emphasis the authors are placing on iron deficiency anaemia, the authors should have at least undertaken peripheral blood morphology evaluation to assess whether there were unique red cell presentations such as pencil-shaped cells. This was not done.
3. Authors should clearly differentiate iron deficiency (ferritin less than 15 ng/dl) from iron deficiency anaemia (ferritin less than 15 ng/dl + Hb below threshold). This distinction has not been made. Since the authors make the erroneous assumption that IDA is equivalent to anaemia, it is not clear what the ROC analyses sought to predict.
4. Moreover, authors write that “Due to being an acute phase protein, ferritin is sensitive to inflammation. Values above 120 ug/L were excluded from the analysis.” The CRP cut-off of (5 mg/l) should be used to screen all the data but not only participants with ferritin levels >120 mcg/L. It is even possible that some with ferritin levels >120 mcg/L had haemochromatosis instead of inflammation-induced increased ferritin levels. Therefore, the assumption of excluding data only based on the ferritin levels >120 mcg/L is erroneous and should be corrected.

**Results**

1. The authors write that “123 women (40.2%) had ferritin values 125 above 15 g/dL using the automated analyzer”. What does this represent? ID or anaemia? The two are not equivalent and must be acknowledged as such. The haematotology anaelyser is not used to estimate ferritin.
2. Again, in line 125 – 127, the authors write “Iron deficiency anemia is characterized by microcytic and hypochromic red blood cells. Out of the sample, 53.95% had microcytosis while hypochromia based on MCH was found in 37.5%.” This is a faulty assumption that only iron deficiency causes hypochromic-microcytic picture. Please revise to correct this.
3. Figures 1 and 2 appear the same; can the authors clarifies the differences? All the figures in panel 1 are the same as that in panel 2.
4. Table 1, 2 and 3, as well as figure 1 should be re-labelled taking into consideration that the sampling sites were different i.e. capillary (Haemocue) vs venous (automated analyser). This different sampling sites definitely leads to differences in the values recorded.
5. The axis labelling on the figure 3 suggest that Haemocue Hb – aHb = 0.36; however, the authors write in line 170 – 171 that “The bias between measurements is 0.36 ± 0.93 g/dL with the automated Hb yielding higher values”. If the data actually represents what the authors have stated, then the y-axis labelling should be revised.
6. In line 175 – 180, what precisely is the ROC analyses seeking to achieve?

**DISCUSSION**

1. I am unable to objectively assess the discussion since the faulty assumptions used for the data analyses has been carried into the data interpretation.
2. For example, in line 193 – 194, the authors write “classifying those with Hb values close to the threshold (12 g/dL). Women with ferritin values above 100 ug/L which generally excludes iron deficiency anemia [24] were regarded as anemics when using the POC device.” And based this on the Haemocue Hb estimation.
