# Supplementary material for: Accuracy of HemoCue301 portable hemoglobin analyzer for anemia screening in capillary blood from women of reproductive age in a deprived region of Northern Peru: An on-field study
Source: PLoS One. 2023 Nov 14;18(11):e0293984. doi: 10.1371/journal.pone.0293984 (PMC10645325; doi:10.1371/journal.pone.0293984)
Supplement: Attachment — Submitted filename: Answer to reviewers_1.docx [file pone.0293984.s003.docx]

**Answer to reviewers**

**Reviewer 1**

**General comment**

**Alarcón-Yaquetto et al have attempted to add data to the venous vrs capillary haemoglobin**

**comparison study viz-a-viz different devices/instrument conundrum. However, the authors have**

**not clearly defined basic concepts well to enable the reader to properly situate the findings. For**

**example, the title is a bit misleading considering the study design employed. In line 86 – 88**

**(page 5), the authors write that “Briefly, 301 cuvettes were filled with a drop of blood from the**

**middle finger fingertip. The first 2 drops of blood were discarded. The microcuvette was put in**

**the cuvette holder and a measurement was done immediately”. In line 90 – 91 (page 5), the**

**authors write that “Upon recruitment, a trained phlebotomist took venous samples of each**

**woman with the BD vacutainer system”. The laboratory samples were venous blood samples.**

**Therefore, the study compared venous haemoglobin (from automated analyser) to capillary**

**haemoglobin (Hemocue 301). it is a known fact that venous haemoglobin and capillary**

**haemoglobin are not equivalent (https://www.mdpi.com/2075-4418/12/12/3191;**

**10.1371/journal.pone.0278350). The title of the study is thus misleading and should be modified**

**to reflect exactly what was done. Again, the distinction between iron deficiency anaemia and**

**anaemia has not been made; the authors give the impression that the two terms are synonymous.**

**However, that is not a scientific fact. This has confounded the write up and the data analyses as**

**well as its interpretation. The manuscript should thus be revised holistically to address these.**

Answer: Thank you for your comments. We have modified the title and we agree, one of the biggest problems in Peru is the generalisation of anaemia as IDA, we made changes throughout the document to reflect the distinction between anaemia and IDA.

**Abstract**

**1. The abstract has been written on the basis of the assumptions that were used to analyse**

**the data. As suggested above and below, the abstract need a re-work after taking the**

**suggested revisions into consideration.**

Answer: The abstract has been modified accordingly.

**2. In the objective statement, indicate that the sampling site was different for the Haemocue**

**and the automated haematology analyser.**

Answer: We apologise for the confusion. The sampling site was the same. Once a participant fitted eligibility criteria and had a fingerstick haemoglobin reading between 8 and 12 g/dL, a venous sample was taken from her at the same site. We transported the samples to the laboratory where further tests were analysed. We clarified this on the manuscript.

**Introduction**

**1. Line 40 – 41, authors seem to portray IDA as being equivalent to anaemia by the**

**statement that “Peru is one of the countries where IDA is a severe public health problem**

**as shown by national statistics.”**

Answer: We have rephrased the paragraph. This is how anaemia is treated in Peru. Even when only point of care devices such as the hemocue are used to diagnose the condition, iron supplementation is given. Confirmatory tests are hardly ever offered to patients living at remote areas.

**2. Line 56 – 57, authors write “Handheld devices have been routinely used to screen for**

**IDA in LMIC countries such as the HemoCue system” to imply that Hemocue is a device**

**for undertaking iron studies. This is misleading since the device estimates haemoglobin**

**levels which is different from ferritin, iron, TIBC etc.**

Answer: We have changed IDA for anaemia.

**3. Authors again write that “The main objective of this study is to assess the suitability and**

**accuracy of this device as a method for IDA diagnosis in women of reproductive age in a**

**coastal city with resource constraints” Hemocue is not meant for iron studies.**

Answer: We have changed IDA for anaemia and emphasise than even when hemocue is not meant for iron studies, it is used as such in Peru as it is the main device used in nation wide field studies to get national rates of anemia which in turn drive health policies.

**Exclusion criteria**

**1. When authors write “Exclusion criterion was amenorrhea non explained by pregnancy**

**and heavy smoking” does this mean that both conditions should be present before**

**exclusion?**

Answer: No. Both were different criteria to exclude. We have rephrased the paragraph.

**Statistical analyses**

**1. Authors defined Microcytosis using the CBC reported mean corpuscular values (MCV) &lt;**

**80 fl.,. and hypochromia by mean corpuscular hemoglobin (MCH) values below 27. There are**

**many causes of microcytic-hypochromic red blood cell picture. With the emphasis the**

**authors are placing on iron deficiency anaemia, the authors should have at least**

**undertaken peripheral blood morphology evaluation to assess whether there were unique**

**red cell presentations such as pencil-shaped cells. This was not done.**

Answer: The CBC analyses followed the ISLH´s guidelines. Briefly, slide review was performed in to assess morphological changes of the red blood cell. No thalassemia nor other type of hemoglobinopathies were found. Historically, prevalence of these type of genetic disorders is very low in Peru. We took measures to exclude subjects with underlying conditions and excluded subjects with inflammation according to proxies. We have updated information in the manuscript to reflect this information.

**3. Authors should clearly differentiate iron deficiency (ferritin less than 15 ng/dl) from iron**

**deficiency anaemia (ferritin less than 15 ng/dl + Hb below threshold). This distinction has**

**not been made. Since the authors make the erroneous assumption that IDA is equivalent**

**to anaemia, it is not clear what the ROC analyses sought to predict.**

Answer: Thank you for your suggestion. We have rephrased the methods section accordingly.

**4. Moreover, authors write that “Due to being an acute phase protein, ferritin is sensitive to**

**inflammation. Values above 120 ug/L were excluded from the analysis.” The CRP cut-off**

**of (5 mg/l) should be used to screen all the data but not only participants with ferritin**

**levels &gt;120 mcg/L. It is even possible that some with ferritin levels &gt;120 mcg/L had**

**haemochromatosis instead of inflammation-induced increased ferritin levels. Therefore,**

**the assumption of excluding data only based on the ferritin levels &gt;120 mcg/L is**

**erroneous and should be corrected.**

Answer: We have now included sensitivity analysis using CRP value of 5 mg/L as suggested.

**Results**

**1. The authors write that “123 women (40.2%) had ferritin values 125 above 15 g/dL using**

**the automated analyzer”. What does this represent? ID or anaemia? The two are not**

**equivalent and must be acknowledged as such. The haematotology anaelyser is not used**

**to estimate ferritin.**

Answer: Thank you for noticing the mistake. 123 women had automated hemoglobin values above 12 g/dL. We meant to provide how this number compared to low ferritin values. We have updated the paragraph and now provide correct percentages.

**2. Again, in line 125 – 127, the authors write “Iron deficiency anemia is characterized by**

**microcytic and hypochromic red blood cells. Out of the sample, 53.95% had microcytosis**

**while hypochromia based on MCH was found in 37.5%.” This is a faulty assumption that**

**only iron deficiency causes hypochromic-microcytic picture. Please revise to correct this.**

Answer: While its true that iron deficiency is not the sole cause of hypochromic and microcytic red blood cells, the other causes are not prevalent in Peru. As we have updated in the methods section, thalassemia was checked as per ISLH guidelines. We have rephrased the paragraph and added in the discussion a sentence noting that iron deficiency is not the sole cause of hypochromic and microcytic RBCs.

**3. Figures 1 and 2 appear the same; can the authors clarifies the differences? All the figures**

**in panel 1 are the same as that in panel 2.**

Answer: The figures have been updated.

**4. Table 1, 2 and 3, as well as figure 1 should be re-labelled taking into consideration that**

**the sampling sites were different i.e. capillary (Haemocue) vs venous (automated**

**analyser). This different sampling sites definitely leads to differences in the values**

**recorded.**

Answer: We have updated the labelling as suggested.

**5. The axis labelling on the figure 3 suggest that Haemocue Hb – aHb = 0.36; however, the**

**authors write in line 170 – 171 that “The bias between measurements is 0.36 ± 0.93 g/dL**

**with the automated Hb yielding higher values”. If the data actually represents what the**

**authors have stated, then the y-axis labelling should be revised.**

Answer: Thank you. We have corrected the y-axis label.

**6. In line 175 – 180, what precisely is the ROC analyses seeking to achieve?**

Answer We sought to analyse the accuracy of both methods in identifying a woman with ferritin values below 15 ng/dL. While the Hemocue is not meant for iron studies, that is the way it is used in Peru. This ROC analysis shows that the way the Peruvian government is tackling anemia by providing universal iron supplementation is an oversimplification of the condition. We run this analysis in the whole sample and in the sample excluding those with CRP values above 5 mg/l as suggested.

**DISCUSSION**

**1. I am unable to objectively assess the discussion since the faulty assumptions used for the**

**data analyses has been carried into the data interpretation. For example, in line 193 – 194, the authors write “classifying those with Hb values close**

**to the threshold (12 g/dL). Women with ferritin values above 100 ug/L which generally**

**excludes iron deficiency anemia [24] were regarded as anemics when using the POC**

**device.” And based this on the Haemocue Hb estimation.**

Answer: We have clarified the paragraph in the sense of noting Hemocue is a screening device and is not meant for iron deficiency diagnosis. However, that is the way it is being used in Peru.

**Reviewer 2**

**Summary/observation**

**The study is very significant as it addresses an important issue in public health. The study aimed to assess the suitability and accuracy of a portable device called HemoCue301 which is used to classify anemia. While the study appreciates the use of HemoCue301, it advised that, the used of the device for the classification of iron deficiency anemia (IDA) should be done with caution.**

Answer: Thank you for your comments.

**Introduction**

**1. Authors should kindly take note and proofread the writing. Example, authors may take a second**

**look at line 56 and 57 of page 3**

Answer: The mentioned paragraph has been edited for clarity.

**Method**

**2. No justification was provided for the exclusion of heavy smokers from the study, line 82 page 5.**

Answer: Smoking is associated with decreased hemoglobin levels although the exact physiological mechanism is not fully understood. It might be due to the compound effect of inflammation, oxidative stress and the alteration of the antithrombotic system (Malenica et al 2017). The WHO has different cut-off values to define anemia in smokers. We have updated the information in the methods section.

Malenica M, Prnjavorac B, Bego T, Dujic T, Semiz S, Skrbo S, Gusic A, Hadzic A, Causevic A. Effect of Cigarette Smoking on Haematological Parameters in Healthy Population. Med Arch. 2017 Apr;71(2):132-136. doi: 10.5455/medarh.2017.71.132-136.

**3. How were the blood samples transported to the laboratory where automated analyzer was**

**used? Line 90 to 96 of page 5**

Answer: We have detailed how samples were transported.

**4. Quality control issues were not indcated**

Answer: We updated the information.
