# Supplementary material for: Accuracy of HemoCue301 portable hemoglobin analyzer for anemia screening in capillary blood from women of reproductive age in a deprived region of Northern Peru: An on-field study
Source: PLoS One. 2023 Nov 14;18(11):e0293984. doi: 10.1371/journal.pone.0293984 (PMC10645325; doi:10.1371/journal.pone.0293984)
Supplement: Attachment — Submitted filename: Replies for PONE-D-23-21513R1.docx [file pone.0293984.s005.docx]

**Replies**

Summary

The authors have made attempt to address the issues that were raised in the initial review process. While the effort is commended, there are still some issues that the authors need to address to make the manuscript technically sound. Authors still make it look like IDA is equivalent to anaemia. While this might be an accepted misconception in Peru (as the authors claim), the manuscript is written for the global audience and should therefore be approached as such. Additionally, the study design was such that fingertip blood (capillary blood) was used for the Hemocue Hb estimation; venous blood was used for the automated analyser Hb estimation. This should be consistently maintained throughout the manuscript to provide context for the data interpretation since the two blood samples are not equivalent.

ABSTRACT

Methods: in the sentence (line 22) “Adult women with HemoCue 301 readings below 12 g/dL were recruited”, authors should indicate that this was capillary blood.

**Reply:** Thank you. We have modified the sentence as suggested.

**In line 26 (Results), authors should specify that capillary blood was used for Hemocue Hb in the sentence “**The Hemocue 301 has a bias of 0.36 ± 0.93 g/dL respect to the automated Hb.”

**Reply:** We have modified the sentence as suggested.

INTRODUCTION

Paragraph 2 of the introduction, authors correctly use the public health significance of anaemia in their classification (41 – 43). However, in paragraph 3 (line 44 – 45), the authors introduce a dimension that the severe anaemia challenge (prevalence of 40% in Peru) is questionable on the supposition that haemoglobin measurement is only a proxy for IDA. IDA classification is based on iron parameter estimates but not solely on haemoglobin measurement. Authors should decouple these in the write up.

**Reply:** We have added a line stating that IDA diagnosis needs the estimation of iron parameters.

The reference 9 cited in the statement “Furthermore, automated hematology analyzers —the method suggested by the WHO [9]” line 54 is not a WHO policy document; please correct this.

**Reply:** Thank you for noticing the error. The correct reference is given in the updated version.

METHODS

The study design was such that fingertip blood (capillary blood) was used for the Hemocue Hb estimation; venous blood was used for the automated analyser Hb estimation. This should be consistently maintained throughout the manuscript to provide context for the data interpretation since the two blood samples are not equivalent. Therefore, under the inclusion criteria, authors should specify in line 80 – 81 that the Hemocue Hb was estimated from capillary blood. Under laboratory analyses (line 95), authors should specify that CBC was undertaken using venous blood. In line 96, the reference 18 as stated by the authors in the main reference list is not an ISLH guideline; please rectify this.

**Reply:** Line 80-81, 95 and the ISLH reference have been modified/added.

Under laboratory analyses (line 96 – 97), authors seem to make an erroneous claim that peripheral blood evaluation alone can be used rule out thalassaemia or other types of haemoglobinopathies. This is haematologically inaccurate. Thalassaemia trait could be silent and may present with few target cells only in peripheral smear. These same target cells are present in iron deficiency, haemoglobin C disease etc. authors should correct this as a peripheral blood morphology evaluation cannot be used to definitively rule out thalassaemia or other haemoglobinopathies.

**Reply:** The paragraph has been rephrased and we added in the limitation section the fact that blood morphology evaluation cannot be used to rule out hemoglobinopathies.

**Statistical analyses**

In line 118 – 119, authors are not clear as to what the ROC sought to determine. The accuracy in detecting what exactly? is this ferritin <15? From the manuscript as a whole, one get the impression that the ROC was undertaken to provide a cut-off for Hemocue Hb or analyser Hb that could predict higher likelihood of IDA. If this is what was the target, the authors need to set out all the assumptions made under the statistical analyses.

**Reply:** The accuracy to detect values <15. We have updated this in the article. We updated methods about estimation of optimal cut-off value.

**RESULTS**

In line 141, in writing “We further classified participants based on tertiles of both HemoCue 301 and automated Hb”, authors should specify that capillary blood was used for Hemocue Hb vs venous blood for analyser Hb.

**Reply:** We have updated the line as suggested.

In table 2, authors should include in the caption capillary blood (Hemocue Hb) and venous blood (analyser Hb).

**Reply:** We have updated the table as suggested.

In line 183, specify the venous vs capillary blood in the sentence.

**Reply:** We have updated the line as suggested.

In line 189 – 196, the ROC & AUC should have a cut-off to guide clinical utility. In addition, there should be other parameters like PPV, NPV, sensitivity and specificity. These are better captured in a separate Table to inform the reader of the reliability of the estimates.

**Reply:** We have added table 4 which provides PPV, NPV, sensitivity and specificity. We updated the methods section respectively.

DISCUSSION

The discussion is silent on perhaps the key highlight of the study; the ROC and AUC data which sought to establish a cut-off for Hemocue Hb and analyser Hb that could be used as a surrogate means of diagnosing IDA in a resource poor setting. The rational for undertaking AUC and cut-off for analytes particularly in poor settings is to provide clinicians with surrogate estimates that provide high degree of suspicion so that treatment could be prescribed even in the absence of specific biochemical tests for diagnosing IDA. However, this important diagnostic dimension of the data is not explored in the discussion which leaves an obvious question as to why the authors employed the ROC in the first place.

**Reply:** We have added table 4 results in the discussion.

Additionally, authors should not be silent on the fact that there are two dimensions on the results; 1) differences in methods [Hemocue vs automated analyser] and 2) different sampling sites of blood [capillary blood for Hemocue Hb vs venous blood for analyser Hb]. These two dimensions should be kept in focus as each affect the results. Should another lab repeat this study by maintain the two measurement techniques (Hemocue vs analyser) but use the same source of blood, the results will not be directly comparable to this study. Thus, it is important that authors stress these in the discussion.

**Reply:** We have updated the discussion accordingly.

MINOR

Line 95 “blood count (CBC) was performed withing two hours of the sample obtention” please correct “withing” and “obstention” and these do not exist.

Line 238 the deploy in “Another argument used for the widespread deploy of HemoCue” to deployment.

**Reply:** We have amended the referenced lines.
